# Urinary TIMP2 and IGFBP7 as early biomarkers of acute kidney injury in septic and nonseptic critically ill patients

**DOI:** 10.1186/cc14368

**Published:** 2015-03-16

**Authors:** M Cuartero, A Betbesé, J Sabater, J Ballús, J Ordóñez

**Affiliations:** 1 Hospital de la Santa Creu i Sant Pau, Universitat Autònoma de Barcelona, Spain; 2 Hospital Universitari Bellvitge, Hospitalet Llobregat, Barcelona, Spain

## Introduction

Sepsis and acute kidney injury (AKI) have a high prevalence in the ICU population. The aim of this study is to describe the composite of tissue inhibitor of metalloproteinases-2 (TIMP2) and insulin-like growth factor-binding protein-7 (IGFBP7) as novel urinary renal biomarkers in both septic and nonseptic patients.

## Methods

We conducted a prospective, observational study in two university hospitals. Patients were admitted in ICU either from the emergency department or after undergoing an acute surgery at hospital admission. Two months prior to the admission, recruited patients had not been admitted to hospital. We collected epidemiological, clinical and laboratory data at admission, 24 and 48 hours. TIMP2*IGFBP7 was analysed in urine samples by a point-of-care device (Nephrocheck®; Astute Medical).

## Results

**Figure 1:**
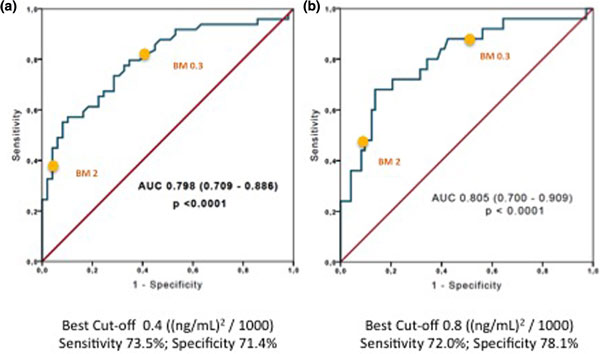
ROC curve and area under the curve (AUC) for the worst [TIMP2]*[IGFBP7] value within the first 48 hours of ICU admission to predict **(a) **AKI and **(b) **AKIN KDIGO ≥2.

The sample included 98 patients (65 men) with mean age 55 ± 17.3 years, length of ICU stay 11.1 ± 14.6 days. In total, 41.4% had sepsis at ICU admission; 59.2% were diagnosed of sepsis within the first 48 hours of stay. We stratified patients based on the presence of AKI as per the AKIN KDIGO definition, as well as their worst level of TIMP2*IGFBP7 during their first 2-day stay. Values of mean and 25th to 75th percentile for the worst value of TIMP2*IGFBP7 were 0.24 (0.11 to 0.46), 0.50 (0.28 to 1.24), 0.94 (0.34 to 3.28) and 3.34 (1.47 to 6.22) for no AKI, AKIN I, II and II respectively (*P *< 0.0001). The worst values for no AKI/no sepsis, no AKI/sepsis, AKI/no sepsis and AKI/sepsis were 0.21 (0.10 to 0.4), 0.32 (0.15 to 0.63), 1.05 (0.41 to 2.31) and 0.98 (0.36 to 3.94) respectively, with *P *< 0.05 for AKI and *P *= NS for sepsis. The AUC ROC curve for prediction of AKI of the worst value was 0.80 with sensitivity of 73.5% and specificity of 71.4% (*P *< 0.0001). In contrast to the Sapphire study, in our population cutoff values of 0.4 and 0.8 (ng/ml)^2^/1,000 predict AKI and AKIN ≥II respectively, regardless of the presence of sepsis. See Figure 1.

## Conclusion

TIMP-2 and IGFBP-7 can predict AKI in both septic and nonseptic critically ill patients. Further pragmatic randomised controlled trials are needed to prove their role on clinical basis.
